# Egr2-dependent microRNA-138 is dispensable for peripheral nerve myelination

**DOI:** 10.1038/s41598-018-22010-8

**Published:** 2018-02-28

**Authors:** Hsin-Pin Lin, Idil Oksuz, John Svaren, Rajeshwar Awatramani

**Affiliations:** 1 Department of Neurology and Center for Genetic Medicine, Northwestern University Feinberg School of Medicine, Chicago, IL 60611 USA; 2 Waisman Center and Department of Comparative Biosciences, University of Wisconsin-Madison, Madison, WI 53705 USA

## Abstract

Recent studies have elucidated the crucial role for microRNAs in peripheral nerve myelination by ablating components of the microRNA synthesis machinery. Few studies have focused on the role of individual microRNAs. To fill this gap, we focused this study on miR-138, which was shown to be drastically reduced in *Dicer1* and *Dgcr8* knockout mice with hypomyelinating phenotypes and to potentially target the negative regulators of Schwann cell differentiation. Here, we show that of two miR-138 encoding loci, mir-138-1 is the predominant locus transcribed in Schwann cells. mir-138-1 is transcriptionally upregulated during myelination and downregulated upon nerve injury. EGR2 is required for mir-138-1 transcription during development, and both SOX10 and EGR2 bind to an active enhancer near the mir-138-1 locus. Based on expression analyses, we hypothesized that miR-138 facilitates the transition between undifferentiated Schwann cells and myelinating Schwann cells. However, in conditional knockouts, we could not detect significant changes in Schwann cell proliferation, cell cycle exit, or myelination. Overall, our results demonstrate that miR-138 is an Egr2-dependent microRNA but is dispensable for Schwann cell myelination.

## Introduction

MicroRNAs (miRNAs; miRs) are small non-coding RNAs that regulate global gene expression in various developmental, physiological and diseased scenarios. In general, these non-coding RNAs are transcribed as long pri-miRNAs, processed by the Microprocessor complex (formed by DROSHA and DGCR8) into pre-miRNAs, exported to the cytoplasm, and cleaved by DICER into mature ~22 nucleotide microRNAs. Each microRNA can silence hundreds of target genes by complementary base pairing with mRNA, which results in translational repression and mRNA destabilization. While individual targets are subtly regulated by each microRNA, the additive effect of coordinated regulation of various biological pathways and feedforward or feedback loops could potentially result in strong phenotypic outputs^1–3^.

Our group and others have knocked out either *Dicer1* or *Dgcr8*, enzymes that are necessary for the biogenesis of the majority of the microRNAs, and found that DICER1 and DGCR8 are crucial in the regulation of Schwann cell (SC) myelination^4–9^. Mice with SC lacking *Dicer1* (*P0*::*Cre*^+^, *Dicer1*^flox/flox^ or *Dicer1* cKOs) or *Dgcr8* (*P0*::*Cre*^+^, *Dgcr8*^flox/flox^ or *Dgcr8* cKOs) during early development partially phenocopy *Egr2*-deficient mice^4,10^ and display severe neurological impairment. In these mutants, the majority of SC stall at the promyelinating stage, unable to myelinate, and some radial sorting defects are also observed. SC lacking either *Dgcr8* or *Dicer1* display decreased EGR2 and increased *Sox2*, *Jun*, and *Notch*^4,6,11^, three well-studied negative regulators of myelination^10,12–14^. These studies led us to posit that microRNAs normally repress negative regulators of differentiation like *Sox2* and *Jun* in order to promote the transition between differentiation states. In addition, in *Dgcr8* cKO nerves, injury specific genes such as *Shh* and *Gdnf* are induced, suggesting that microRNAs may repress the injury-related gene expression program^11^.

While conditional knockouts of *Dgcr8* and *Dicer1* have revealed the general importance of microRNAs in SC differentiation, a logical next step would be to pinpoint the role of individual microRNAs in this process. To date, few studies have revealed the role of individual microRNAs in SC *in vivo*. One recent study suggested that the let-7 family of microRNAs suppresses *Notch* signaling and promotes EGR2 expression and myelination^15^. Here we sought to fill this gap, focusing on miR-138. In oligodendrocytes, miR-138 has been identified as a pro-differentiation factor^16^. In SC, miR-138 levels have previously been shown to be significantly upregulated during development and drastically reduced in the sciatic nerves of *Dicer1* cKOs and *Dgcr8* cKOs^4,11^. miR-138 is predicted to target several immature SC mRNAs and negative regulators of myelination, including *Ccnd1*, *Sox2* and *Jun*, although the functional effects in luciferase assays were modest^4,17^. In line with gene expression and bioinformatic analyses, we hypothesized that EGR2 induces transcriptional upregulation of miR-138, and miR-138 helps EGR2 to downregulate the negative regulators, driving myelination forward. To test this, we evaluated the expression of miR-138, its dependence on *Egr2*, and the impact of its loss of function in SC.

## Results

### miR-138 is downregulated upon nerve injury

**Figure 1:**
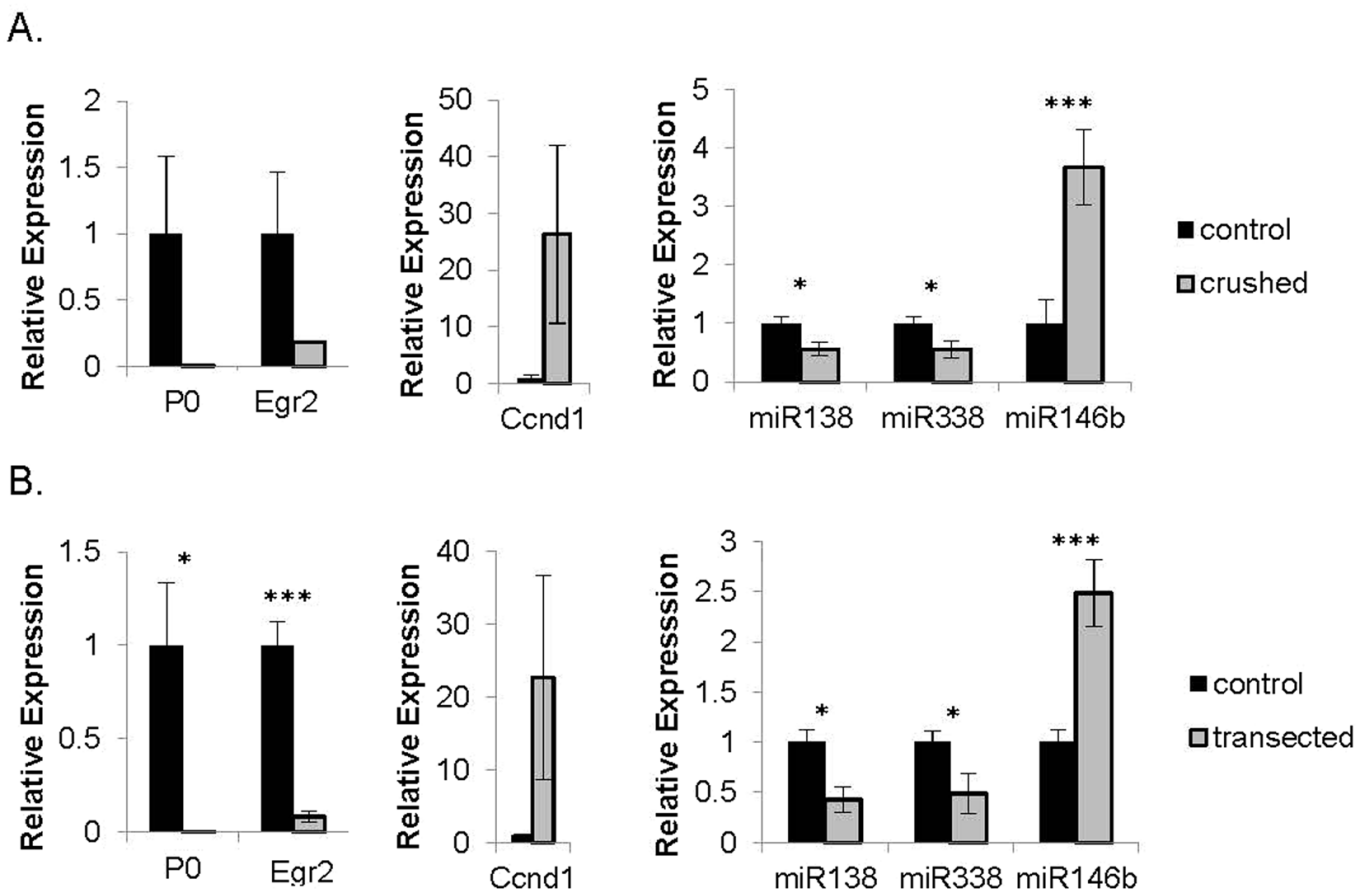
miR-138 is downregulated upon nerve injury. Quantitative RT-PCR expression levels of selected SC genes and miR-138, 338-3p and 146b in injured nerves ((**A**) crushed nerve, (**B**) transected nerve) (gray bars) and contralateral controls (black bars) (*p < 0.05, **p < 0.001, ***p < 0.005). For crushed nerve experiments, sciatic nerves of wildtype mice were harvested five days post-surgery. For transected nerve experiments, sciatic nerves were harvested four days post-surgery.

Our previous study demonstrated that miR-138 is upregulated during postnatal SC development^4^. Here, we sought to examine whether its expression is altered by nerve injury. Upon sciatic nerve injury, the SC dedifferentiation program is activated within hours and continues to evolve over days. Myelin genes and the positive regulators of differentiation are downregulated and the negative regulators are upregulated. To test whether our microRNA candidates, like other key positive regulators of myelination, are downregulated during SC dedifferentiation, we performed crush and transection surgery on the sciatic nerves of wildtype mice. Crushed or transected and contralateral control nerves were harvested five days or four days post-surgery, respectively, and quantitative RT-PCR was performed to measure levels of selected SC genes and microRNAs. Expression of myelin gene *Mpz* and *Egr2* was drastically decreased in injured nerves compared to controls, demonstrating the validity of the injury. miR-138 was significantly downregulated in injured nerves (Fig. 1A,B). miR-338-3p, a microRNA with a similar developmental expression profile, was also downregulated. By contrast, miR-146b, a developmentally increased microRNA, was significantly upregulated after injury, possibly due to expression in infiltrating macrophages^18,19^. Thus, at least miR-138 and miR-338-3p expression resemble that of other key regulators in the network, displaying mirror image expression in differentiation versus dedifferentiation situations^20^.

### mir-138-1 is the predominant locus transcribed in SC

**Figure 2:**
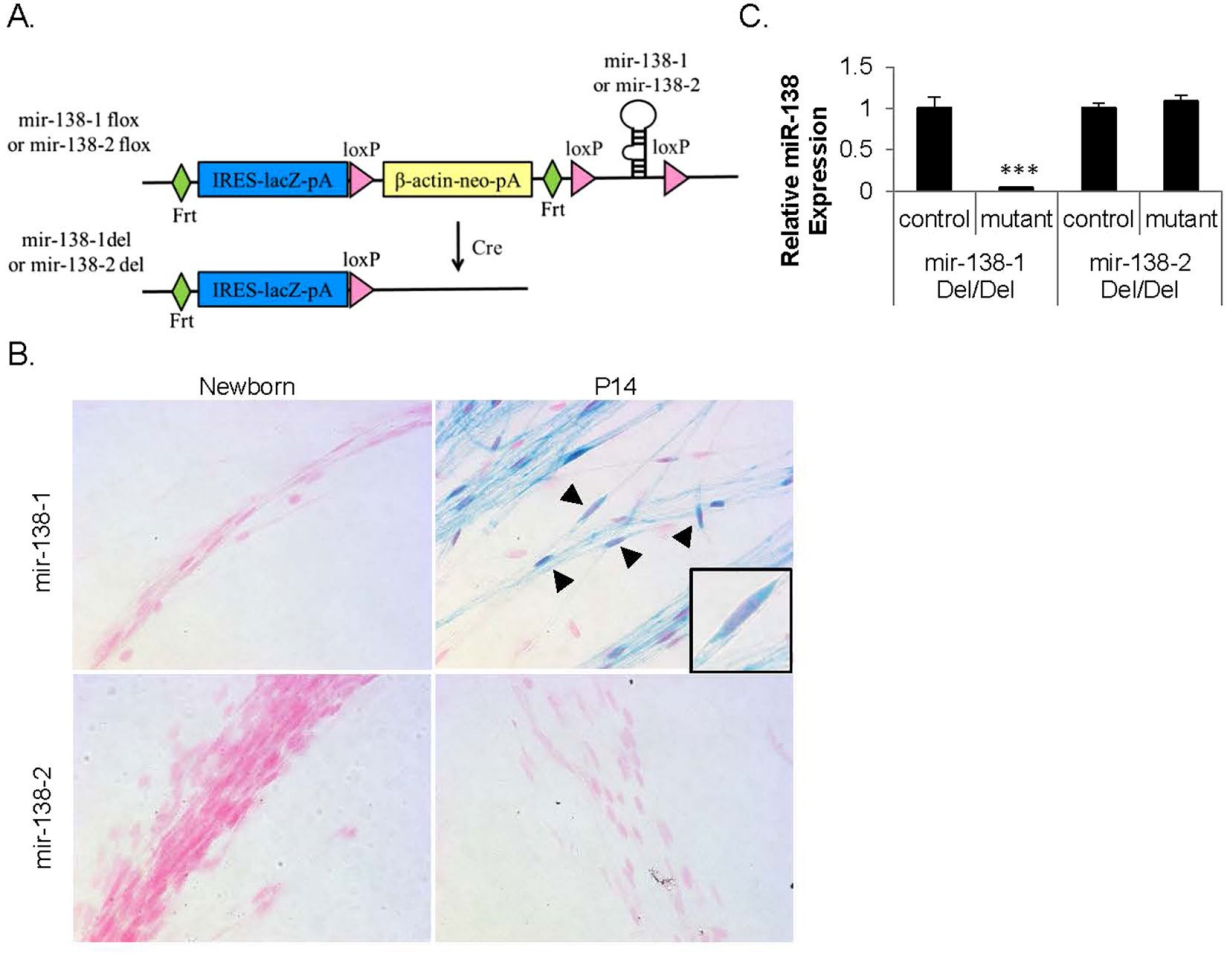
mir-138-1 is the predominant locus transcribed in SC. (**A**) Schematic representation of the microRNA allele. A promoter-less *lacZ* reporter with an internal ribosome entry site (IRES) with a polyA (pA) signal, β-actin-driven neomycin selection marker with a pA signal, and a microRNA stem-loop flanked by *loxP* sites were targeted into the microRNA locus. The mice can be crossed with either germline- or tissue-specific *Cre* transgenic mice. We crossed mir-138-1 mice and mir-138-2 mice with germline deleter *β-actin*::*Cre*^+^ mice to produce mice with a *lacZ*-tagged deleted allele that lacked the *β*-*actin* promoter-neomycin casette (del). (**B**) Teased sciatic nerves of heterozygous *lacZ*-tagged mir-138-1^del/wt^ mice and heterozygous *lacZ*-tagged mir-138-2^del/wt^ mice at newborn and P14, stained with Xgal (blue) and Nuclear Fast Red (pink/ light red) (N ≥ 3). Arrow heads point to SC with oval-shaped nuclei. Insert shows one SC at high magnification. (**C**) miR-138 quantitative RT-PCR of P14 mir-138-1^del/del^ and mir-138-2^del/del^ sciatic nerves. miR-138 level is 28 fold lower in the sciatic nerves of the mir-138-1^del/del^ homozygous mutants than the controls (N ≥ 3, p = 0.0019), while miR-138 level in the mir-138-2^del/del^ homozygous mutants does not significantly differ from the controls (***p < 0.005).

There are two distinct genetic loci, mir-138-1 (chromosome 9) and mir-138-2 (chromosome 8), that may give rise to mature miR-138. To determine which locus is transcribed in SC during development, we obtained mir-138-1^flox/wt^ mice and mir-138-2^flox/wt^ mice from Mutant Mouse Regional Resource Centers (MMRRC). These reporter mice were generated by knocking in a promoterless IRES-*lacZ* with a polyA (pA) signal, *β-actin*-driven neomycin selection marker with a pA signal, and a microRNA stem-loop flanked by *loxP* sites (*FRT-*IRES*-lacZ-*pA*-loxP-β-actin-neo-*pA*-FRT*-*loxP*-microRNA-*loxP*) at the microRNA loci^21^ (Fig. 2A). These mice can be crossed with either germline- or tissue-specific *Cre* transgenic mice to generate a reporter-tagged microRNA knockout allele.

We first crossed these mice with *β-actin*::*Cre*^+^ mice (Jackson Laboratory) in order to remove the *β-actin*-neomycin cassette in the germline and thereby prevent undesired transcriptional interference at these loci. In this configuration, this allele is designed to be a reporter knock-in that reports the transcriptional status of the locus (Fig. 2A). We harvested sciatic nerves from the resulting heterozygous *lacZ*-tagged mir-138-1^del/wt^ mice and heterozygous *lacZ*-tagged mir-138-2^del/wt^ mice at P0 (newborn) and P14. In these mice, cells would produce β-galactosidase and be labeled blue when stained with Xgal if the tagged loci were transcribed. Xgal staining of the teased sciatic nerves showed that SC of *lacZ*-tagged mir-138-1^del/wt^ were labeled blue at P14, the peak of myelin gene expression in SC, but not at P0. SC of *lacZ*-tagged mir-138-2^del/wt^ were not labeled at P0 or P14 (SC are shown with characteristic oval-shaped nuclei, Fig. 2B). This result implies that the mir-138-1 locus is transcribed at P14, but is undetectable by this assay in newborn SC, a pattern consistent with miR-138 levels during development^4^. On the other hand, transcription of the mir-138-2 locus was undetectable in newborn or P14 nerves.

The above results suggest that the mir-138-1 locus is the major contributor to mature miR-138 levels in SC. To confirm this, we performed quantitative RT-PCR on homozygous *lacZ* tagged mutants and littermate controls at P14. miR-138 levels were 28 fold lower in the sciatic nerves of the mir-138-1 mutants compared to controls. By contrast, in the mir-138-2 mutants, miR-138 levels did not significantly differ from the controls (Fig. 2C). These data demonstrate that the mir-138-1 locus is the major contributor to miR-138 levels in SC, although a small contribution from the mir-138-2 locus cannot be ruled out.

### Egr2 is required for mir-138-1 transcription during development and both EGR2 and SOX10 bind to an active enhancer near the mir-138-1 locus

Our previous work had led us to postulate that EGR2 is an activator of key microRNAs that aid in the repression of genes expressed in immature SC^4,11^. To determine whether *Egr2* is required for the upregulation of specific microRNAs, we obtained mice harboring the *Egr2::Cre* allele, wherein *Cre* was placed at the ATG of *Egr2*, thereby creating an *Egr2* null allele^22^. In order to comprehensively evaluate microRNA levels in *Egr2*^−/−^ mice at P5, we subjected control and mutant sciatic nerve samples to measurements by TaqMan Array Rodent MicroRNA Cards. Indeed, the expression of some candidates, such as miR-338 and miR-146b, was reduced in the P5 *Egr2*^−/−^ sciatic nerve. However, when we applied stringent statistical analysis, including adjusting the p-value for false discovery rate with ExpressionSuite software, miR-138 was the only microRNA that was significantly altered (fold change = 0.038 of normal, p-value = 0.042).

**Figure 3:**
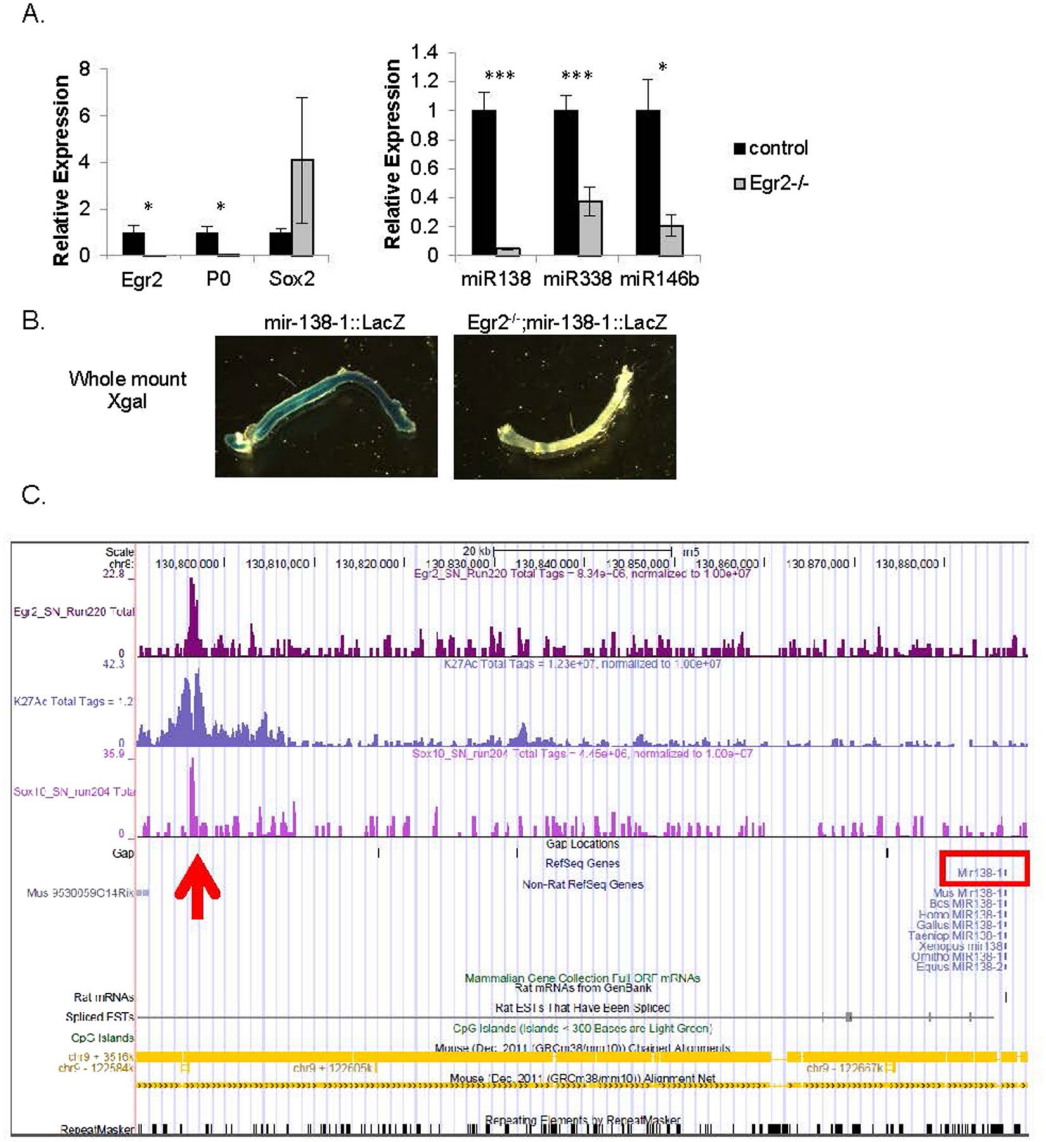
Egr2 is required for mir-138-1 transcription during development and both SOX10 and EGR2 bind to an active enhancer near the mir-138-1 locus. (**A**) Quantitative RT-PCR expression levels of selected SC genes (*Mpz*, *Egr2* and *Sox2*) and microRNA candidates (miR-138, 338-3p and 146b) in *Egr2-*null sciatic nerves (gray bars) and littermate controls (black bars) at P5 (*p < 0.05, **p < 0.001, ***p < 0.005). (**B**) Shown are whole mount Xgal staining of P7 mir-138-1::*LacZ* and *Egr2*^−/−^; mir-138-1::*LacZ* sciatic nerves. Blue Xgal staining reports endogenous transcription activities at mir-138-1 locus. Transcription activity at mir-138-1 at P7 is detectable in control sciatic nerve but is not detectable in *Egr2*^−/−^ sciatic nerves. (**C**) The UCSC genome browser view shown in the figure displays peaks of SOX10 and EGR2 binding based on analysis of the ChIP-Seq data from pooled P15 rat sciatic nerves^25^. Histone H3 K27Ac data is a track of active enhancers also obtained from P15 sciatic nerves. The peaks are co-localized (red arrow) and within 100 kb from mir-138-1 locus (red box).

To confirm this, we profiled sciatic nerves of *Egr2*^−/−^ mice and control littermates with quantitative RT-PCR. As shown in Fig. 3A, levels of myelin gene *Mpz* and positive regulator *Egr2* were decreased while levels of *Sox2* were increased in the sciatic nerves of the *Egr2* null mice, consistent with previous findings^10^. Levels of miR-138 were drastically decreased in *Egr2* null samples. In this assay, we found that levels of miR-338 and miR-146b were also significantly decreased. Thus, EGR2 is either directly or indirectly upstream of miR-138, miR-338, and miR-146b *in vivo*.

The reduction of miR-138 in *Egr2*^−/−^ could be because microRNA processing is affected in this mutant or because miR-138 is a transcriptional target of EGR2. To test whether the upregulation of miR-138 by EGR2 during development is regulated at the level of transcription, we stained whole mount P7 sciatic nerves of *Egr2*^−/−^; mir-138-1::*lacZ* and mir-138-1::*lacZ* control mice with Xgal. As shown in Fig. 3B, Xgal staining was detectable in control sciatic nerves at P7. However, when both copies of *Egr2* were lost, Xgal staining was undetectable. Therefore, EGR2 regulates miR-138 upregulation during development at the transcriptional level. *Egr2*^−/−^ sciatic nerves display very little transcriptional activity at the mir-138-1 locus and consequently, mature miR-138 levels are extremely low in these nerves.

Since EGR2, the master regulatory transcription factor, is known to cooperate with SOX10 and bind directly to promoters and enhancers of many genes encoding key myelin proteins for activation^23,24^, we wanted to know whether EGR2 also directly binds to the promoters and enhancers of our microRNA candidates. Chromatin immunoprecipitation sequencing (ChIP-Seq) analysis of SOX10 and EGR2 in pooled rat sciatic nerves at P15 has been reported^25–29^. Histone H3K27Ac data has also been obtained in rat sciatic nerves at P15^27^. We used the same ChIP-Seq database and found that both SOX10 and EGR2 bound to an enhancer near the mir-138-1 locus marked by histone H3K27Ac (red arrow in Fig. 3C), but did not bind near the mir-138-2 locus (not shown). Histone H3K27ac is known to mark active enhancers (H3K27ac+) but not poised enhancers (H3K27ac−) and can be used to predict developmental state^30^. This binding site likely accounts for EGR2-dependent transcription of the mir-138-1 locus in SC, although mutation of this site would be required to definitively confirm this. miR-138 is also expressed in oligodendrocytes^16^. The same enhancer was found to be occupied by SOX10 and OLIG2 in oligodendrocytes (not shown).

### Sciatic nerves of P4 mir-138-1 cKO mice appear to be morphologically similar to the controls

**Figure 4:**
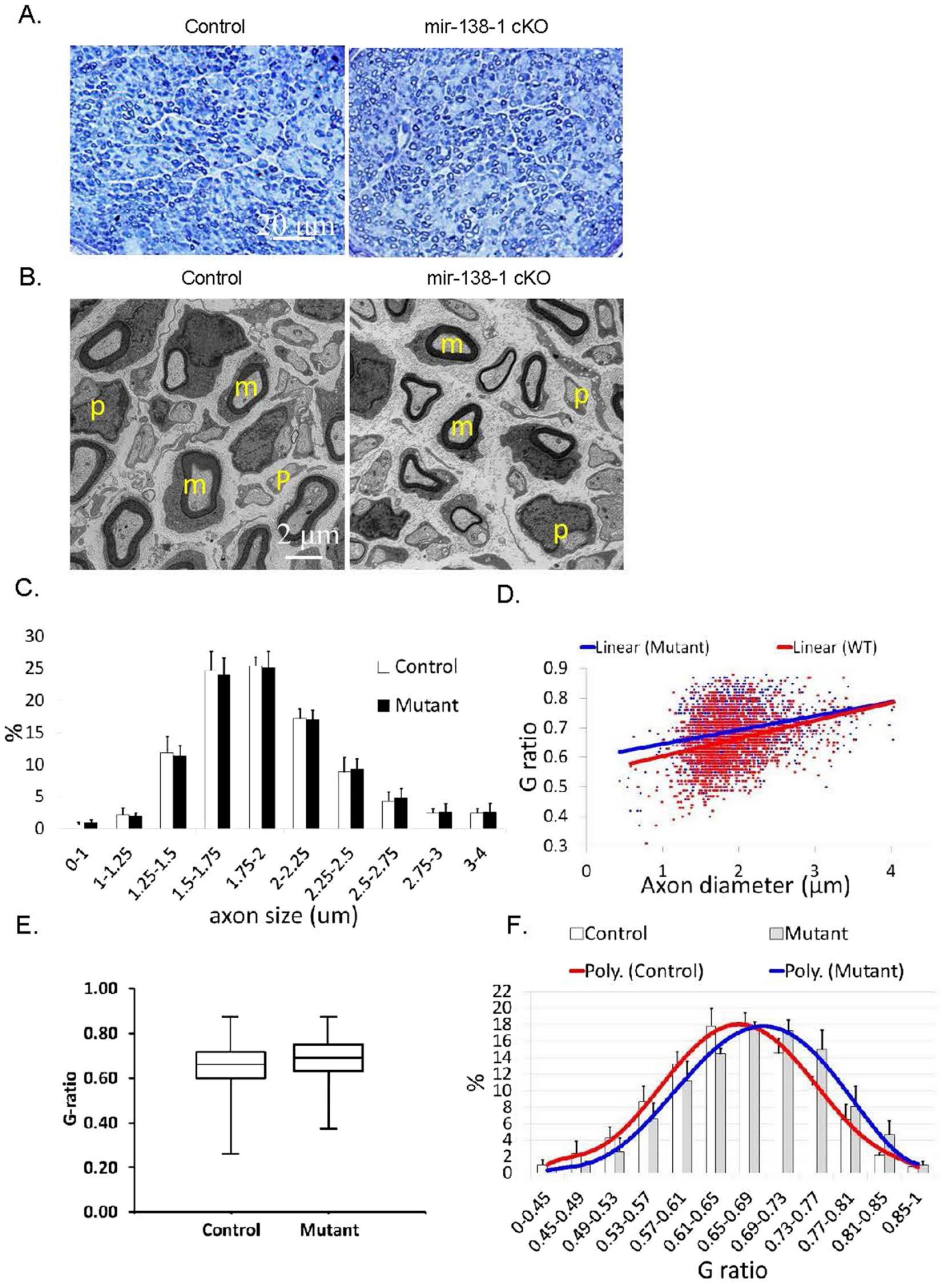
Sciatic nerves of P4 mir-138-1 cKO mice appear to be morphologically similar to the controls. (**A**) Semi-thin cross sections of P4 mir-138-1 cKO and control sciatic nerves stained with Toluidine blue. In both mutants and controls, normal numbers of myelin sheaths are observed in the nerves. (**B**) Electron microscopic analyses of P4 mir-138-1 cKO and control sciatic nerves in cross sections. In both mutants and controls, myelinating SC are mainly observed. (**C**) Percentage (%) of axons in axon diameter ranges (μm). There is no significant difference in axon diameter distribution in P4 control and mir-138-1 cKO nerves. (**D**) Scatter plot of g-ratio (axon diameter/fiber diameter) versus axon diameter. There is no significant difference in the relationship between axon diameter and myelin thickness in P4 control and mir-138-1 cKO nerves. (**E**) Box-and-whisker plot of g-ratios measured from the P4 control and mir-138-1 cKO axons. >400 axons were measured in each sample. (**F**) Percentage (%) of axons in g-ratios ranges. There is no significant difference in myelin thickness distribution between P4 mir-138-1 cKO and control nerves. N = 3 in (**A**–**F**).

To test whether miR-138 is required in SC development, we conditionally removed miR-138 in SC by crossing mir-138-1^flox/flox^ mice to *P0*::*Cre*^+^, mir-138-1^flox/wt^ mice. We harvested P4 sciatic nerves of the resulting *P0*::*Cre*^+^, mir-138-1^flox/flox^ mutants (henceforth called mir-138-1 cKOs) and controls for morphological analysis. Shown are semithin cross sections with toluidine blue staining and electron microscopic cross sections (Fig. 4A,B). Myelination appeared normal in both the mutants and the controls. In both genotypes, the majority of the SC were either promyelinating or myelinating; few were unsorted. In controls, about 48% SC were myelinating, 44% were promyelinating, 7% were unsorted and 1% were non-myelinating. In mir-138-1 cKO mutants, about 44% SC were myelinating, 45% were promyelinating, 10% were unsorted and 1% were non-myelinating (p = 0.5418, 0.8979, 0.2587, 0.8108, respectively). These data indicate that there is no overt SC differentiation deficit in miR138-1 cKO nerves.

Since myelination is still in process at P4, it is possible that a mild delay in myelination could be detected by the presence of thinner myelin sheaths on axons of the same diameter size. Therefore, we measured g-ratios (axon diameter/fiber diameter) and axon diameters of myelinating SC at P4 (Fig. 4C–F). However, scatter plots of g-ratio and axon diameter distribution of myelinating SC did not show any significant differences between the control and the mutant sciatic nerves, indicating that, at early time points, myelin sheaths reached similar thickness on axons of similar sizes in the mutants and the controls. Together, these results suggest that loss of miR-138 in SC during development does not block myelination or significantly alter the timing of myelination in sciatic nerves.

### mir-138-1 cKO sciatic nerves display normal proliferation, cell cycle exit, and cell numbers

**Figure 5:**
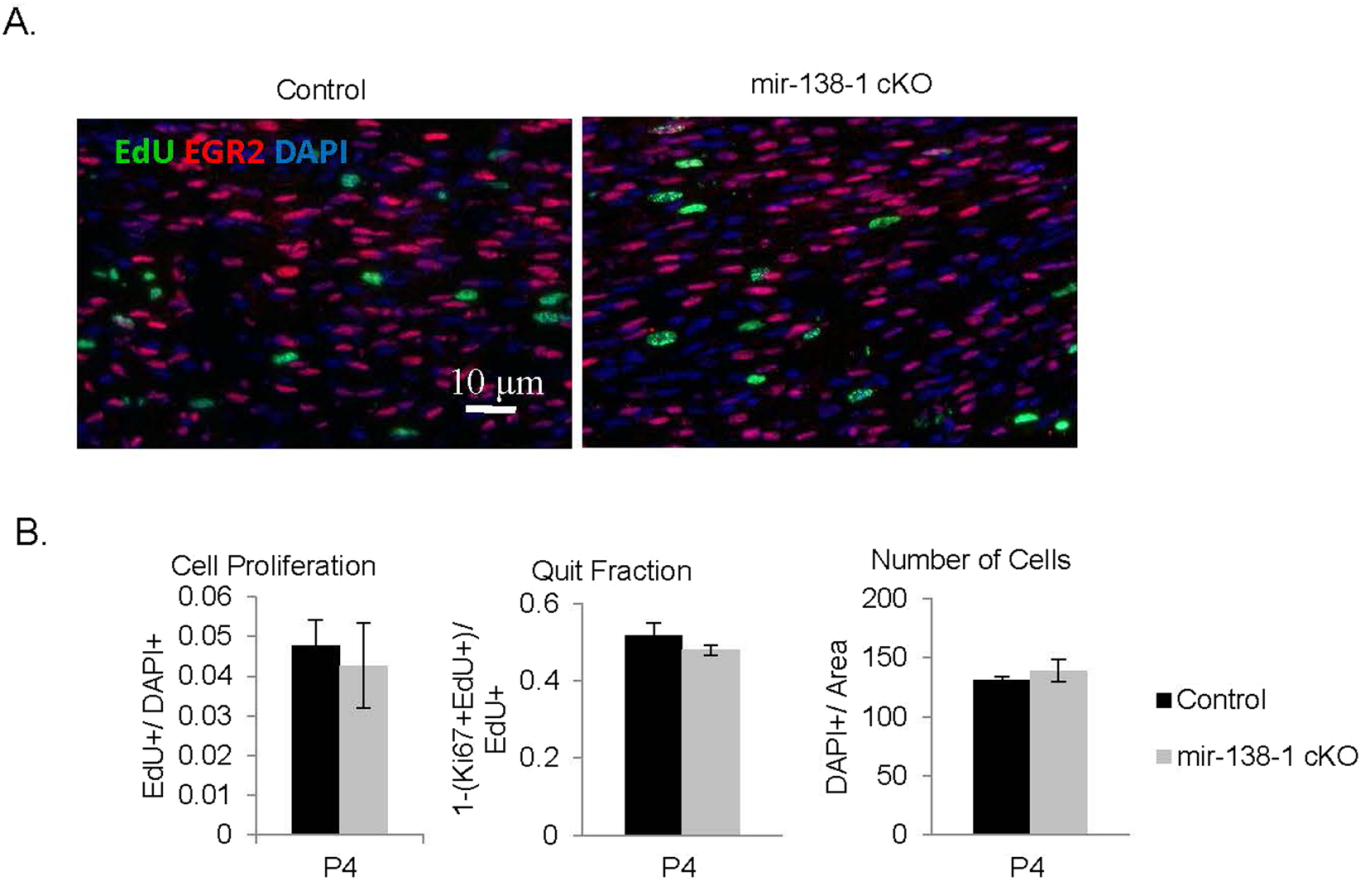
mir-138-1 cKO sciatic nerves do not display alterations in proliferation, cell cycle exit, and in cell numbers. (**A**) EdU (green), EGR2 (red) and DAPI (blue) staining of P4 mutant and control sciatic nerves. (**B**) Quantification of EGR2 expression (EGR2+/DAPI+) and cell proliferation rate (EdU+/DAPI+) in P4, and quit fraction (Ki67-EdU+/EdU+) and cell numbers (DAPI+/area) in P4 mutant and control sciatic nerves. There is no significant difference detected in the number of EGR2-expressing cells (N ≥ 4; P4, p = 0.7847), cell proliferation rate (N ≥ 4; P4, p = 0.6942), quit fraction (N ≥ 4; P4, p = 0.1500), or cell number per unit area (N ≥ 4; P4, p = 0.4910). Black bar = controls. Gray bar = mir-138-1 cKOs.

miR-138 has been shown to be involved in the proliferation of various cell types^31–34^. In SC, miR-138 could potentially repress cell cycle genes such as *Ccnd1*^4,35^. To test the hypothesis that miR-138 may alter proliferation and/or cell cycle exit in SC, we injected mice with EdU for 2 hours, performed immunohistochemistry on harvested sciatic nerves and quantified EdU+/DAPI+ cells at P4 (Fig. 5A). We found no difference in the number of proliferating cells. In controls, 0.048 ± 0.006 EdU+ cells were counted per DAPI + cell. Similarly, in mutants, 0.043 ± 0.011 EdU+ cells were counted per DAPI+ cell (N ≥ 4, p = 0.6942) (Fig. 5B). To check whether loss of mir-138-1 led to defects in cell cycle exit, we injected a pulse of EdU at P4, harvested nerves after 24 hours, and measured the quit fraction (Ki67-EdU+/EdU+). The results showed that, though mir-138-1 mutants seemed to exhibit a lower quit fraction, the difference did not reach statistical significance. Consistent with these analyses, when we performed immunohistochemistry on mir-138-1 cKO sciatic nerves at P4, we found that the number of EGR2+ cells was similar between mutants and controls (Fig. 5A). In controls, 0.354 ± 0.012 EGR2+ cells were counted per DAPI+ cell. Similarly, in mutants, 0.365 ± 0.036 EGR2+ cells were counted per DAPI+ cell (N ≥ 4, p = 0.7847) (Fig. 5B). Finally, we also measured total cell numbers (DAPI+/area) at P4 (Fig. 5B) and found no significant difference between mutants and controls. Loss of mir-138-1 does not appear to alter proliferation, cell cycle exit, or cell numbers.

### Sciatic nerves of adult mir-138-1/-2 cKO mice also appear to be morphologically similar to controls

**Figure 6:**
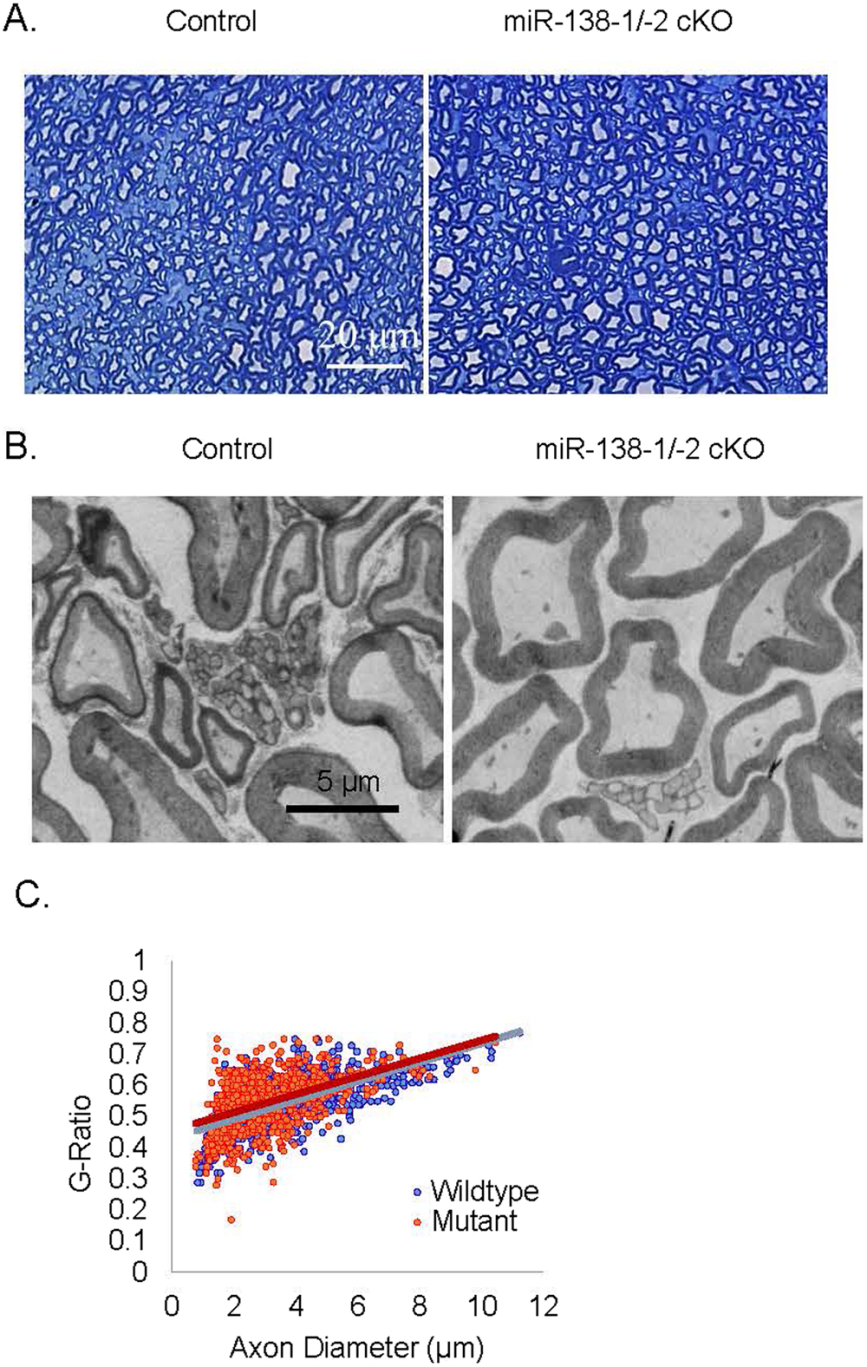
Sciatic nerves of adult mir-138-1/-2 cKOs are myelinated normally. (**A**) Semi-thin cross sections stained with Toluidine blue and (**B**) Electron microscopic photos of sciatic nerves from adult mir-138-1/-2 cKO mice and the littermate controls. In both mutants and controls, myelin sheaths are observed in the nerves. (**C**) Scatter plot of g-ratios and axon diameters shows no significant difference in myelin thickness and axon size relationship between adult mir-138-1/-2 cKO mutants and controls (N = 3).

We considered the possibility that the mir-138-2 locus may contribute very low but significant levels of miR-138 in SC that is sufficient for myelination even in the absence of mir-138-1. To test this possibility, we bred mir-138-1^flox/flox^, mir-138-2^flox/flox^ mice to *P0::Cre*^+^, mir-138-1^flox/wt^, mir-138-2^flox/wt^ mice to generate double knockouts that completely lack miR-138. We observed no obvious behavioral abnormality from the resulting *P0::Cre*^+^, mir-138-1^flox/flox^, mir-138-2^flox/flox^ mutants (mir-138-1/-2 cKOs) at two months old, and semi-thin analysis of adult mir-138-1/-2 cKO sciatic nerve cross sections showed, similar to the controls, normal myelin sheaths visible by dark blue staining of Toluidine blue (Fig. 6A). Figure 6B shows EM cross sections of control and mutant nerves. The scatter plot of their g-ratios and their axon diameter distribution of myelinating SC are shown in Fig. 6C. No significant morphological differences between control and mutant sciatic nerves were detected. Thus, adult mir-138-1/-2 cKOs do not display obvious myelination defects. Based on these analyses, we conclude that miR-138 is largely dispensable for SC myelination.

## Discussion

MicroRNAs have been implicated in a wide spectrum of physiological processes including, among others, cellular differentiation. Recent studies using conditional knockouts of *Dicer1* or *Dgcr8* have implicated microRNAs in CNS and PNS myelination^9,28,36^. Following up these studies, emerging literature points to roles for specific microRNAs in myelination, the best studied examples in mice being miR-219 and miR-338 in oligodendrocyte differentiation and let-7 in SC differentiation^15,16,37,38^. Several other studies have studied the role of miR-21, miR-34, miR-9, miR-132, miR-140, miR-148b-3p, miR-210, miR-29a, miR-709 in SC proliferation and migration *in vitro*^8,17,26,28,39–43^. Here, we examined the expression of miR-138 and its role in SC differentiation. We found that the developmental profile of this microRNA resembles that of a pro-differentiation factor. Extrapolating from findings on the role of microRNAs in oligodendrocyte differentiation^16^, we hypothesized that miR-138 may have a functional role in promoting timely myelination. However, conditionally knocking out miR-138 did not reveal any major myelination deficit.

In humans and mice, miR-138 may be transcribed from the mir-138-1 locus or mir-138-2 locus^44^. These two loci are often differentially transcribed^45^. Consistent with this, we found that the mir-138-1 locus is the predominant source of miR-138 in SC. This locus is developmentally upregulated during myelination and its transcription is dependent on EGR2. Analyzing Chip-Seq data from P15 nerves, we found EGR2 and SOX10 binding sites in the vicinity of the mir-138-1 locus in a region with active enhancer marks, suggesting that EGR2 may directly regulate mir-138-1 transcription. Thus, in addition to EGR2 repression of antecedent gene expression programs via NAB corepressors^46^, EGR2 induction of miR-138, and potentially miR-338 and miR-146b, may be a second mechanism by which EGR2 carries out or reinforces the repression. Additionally, the *Egr2* transcript itself has a miR-138 predicted binding site in the 3′UTR, possibly to modulate levels of EGR2 as part of a feedback mechanism^17^.

We studied the function of miR-138 *in vivo* with loss-of-function experiments, hypothesizing that miR-138 may facilitate the transition of SC from undifferentiated to myelinating states. However, mir-138-1 cKO sciatic nerves displayed normal proliferation, cell cycle exit, and cell numbers. One possible explanation of this result is that miR-138 plays a significant biological role in early SC development and *P0::Cre* deleted miR-138 too late. *P0::Cre* is turned on around E15 in immature SC^47^, but since microRNAs are stable^48^, if there were low levels of miR-138 in early SC, then timing of deletion could make a difference. Using a *Cre* driver that is active earlier in SC development (such as *Dhh::Cre*) may have resulted in a detectable phenotypic effect. Indeed, in a study on *Notch* signaling, *Dhh::Cre*-mediated gene activation elicited slightly more robust phenotypes than *P0::Cre*^14^.

A second possible explanation of the normal myelination of mir-138-1 and mir-138-1/-2 cKO SC is that the potential de-repression of antecedent genes is mild. Mild derepression may not be sufficient to prevent SC differentiation *in vivo*. Indeed, gain-of-function experiments for *Notch* show only a mild delay in myelination despite fairly robust *Notch* overexpression^14^. Likewise, in the case of *Sox2*, homozygous overexpressing mice display a permanent block in myelination, whereas heterozygous overexpressing mice display only a transient delay^13^. In coculture systems, strong lentiviral overexpression of *Sox2* results in a block of myelination^10^. Thus, a mild derepression of negative regulators, even if they do occur, may not yield detectable phenotypes.

A third possible explanation for the lack of phenotype, is that there is genetic redundancy from microRNAs that do not share seed sequences with miR-138 but have overlapping sets of targets^2^. Bioinformatic prediction of microRNA targets generally predict hundreds of targets for each microRNA and dozens of microRNA recognition sites on each mRNA^49^. These highly complex target networks pose a significant challenge to study the mechanism and function of a single microRNA. For example, *Ccnd1* is targeted by miR-138, but it is also targeted by miR-34a, miR-16, miR-195, miR-153, miR-503 and many other microRNAs^50^. As microRNAs often function by fine-tuning the expression of numerous targets, conceptually, it is possible that the phenotypes of certain microRNAs can only be elicited when microRNAs that share several of their targets are also removed from the particular cellular environment^2^.

The majority of individual microRNA conditional knockout mice examined thus far, like our miR-138 cKO, do not exhibit overt developmental phenotypes^2,51^. Rather, the functions of individual microRNAs have only been revealed under stress conditions^52^. Loss of microRNAs may enhance or diminish the organismal response to stress, thereby augmenting or diminishing a pathologic process^2^. For instance, miR-208a conditional knockout mice display little to no effect on cardiovascular development or normal function, but show significantly reduced stress-responsive cardiac hypertrophy in response to pressure overload^53^. It will be interesting to subject mir-138-1 cKO to SC-related stress paradigms such as nerve injury or peripheral neuropathies.

Conditional deletion of genes in SC have sometimes resulted in no developmental phenotype, but a robust phenotype upon nerve injury. For instance, mice with conditional loss of *Jun* in SC, myelinate normally during development but show profound deficits in SC gene expression, and the ability to promote axonal regeneration upon nerve injury^54,55^. It is possible that the functional role of miR-138 may be revealed in an injury paradigm. Since miR-138 expression resembles that of a positive regulator of myelination, we would expect that the loss of miR-138 may delay remyelination after nerve injury. Another way to reveal the function of miR-138 could be by crossing mir-138-1 cKO mice to Egr2^Lo/Lo^ mutant mice^10^; this may exacerbate the hypomyelination phenotype if miR-138 is indeed induced by EGR2 to perform part of the downstream functions of EGR2 as a repressor of antecedent gene programs. Findings from the study of miR-138 conditional knockouts under these physiological stresses are likely to provide new understanding of disease pathophysiology and uncover opportunities for therapeutic intervention.

In conclusion, the relatively normal development revealed by our studies of mir-138-1 cKOs highlights the challenges of dissecting single microRNA functions *in vivo*. Although the first microRNAs discovered, lin-4 and let-7, result in potent developmental defects when deleted^56,57^, it is becoming evident that most individual microRNA knockout mice studied to date display no significant developmental defects. On the contrary, microRNAs seem to play a valuable role in maintaining homeostasis under stress or other pathological disturbances^2^. Adding onto the complicated overlaying networks of hundreds of subtly regulated targets and the drastic mechanistic differences between each microRNA are the challenges of applying the right battery of stress paradigms to reveal otherwise undetectable defects. This process is likely to be demanding, but the new knowledge will be highly applicable to disease pathophysiology and therapeutic intervention.

## Methods

### Generation of Mice

For mir-138-1 and mir-138-2 expression studies, we crossed *β-Actin*::*Cre*^+^ mice (Jackson Laboratory) to mir-138-1^flox/wt^ mice and mir-138-2^flox/wt^ mice obtained from Mutant Mouse Regional Resource Centers (MMRRC)^21^ to obtain heterozygous lacZ-tagged mir-138-1^del/wt^ mice and heterozygous lacZ-tagged mir-138-2^del/wt^ mice. For miR-138 loss-of-function studies, we bred *P0*::*Cre*^+^ ^58^, mir-138-1^flox/wt^ mice to mir-138-1^flox/flox^ mice and *P0::Cre*^+^, mir-138-1^flox/wt^, mir-138-2^flox/wt^ mice to mir-138-1^flox/flox^, mir-138-2^flox/flox^ mice. When necessary, 50 mg/kg EdU was administered through intraperitoneal or subcutaneous (for neonates) injection 2 hours (proliferation analyses) or 24 hours (quit fraction analyses) before dissection. Quit fraction analysis was performed by counting the total number of EdU + cells and the number of EdU + /Ki67 + cells as previously described^59^. All mice were maintained and sacrificed according to the protocols approved by the Northwestern University Animal Care and Use Committee.

### Semithin Analyses and Electron Microscopy (EM)

Sciatic nerve samples were submerged in fixative (0.1 M sodium cacodylate buffer pH 7.3 containing 2% paraformaldehyde and 2.5% glutaraldehyde). Semithin section and electron microscopic analyses were performed at the Center for Advance Microscopy at Northwestern University Feinberg School of Medicine. Samples were post-fixed with 2% osmium tetroxide in 0.1 M sodium cacodylate buffer, rinsed with distilled water, en bloc stained with 3% uranyl acetate, rinsed with distilled water, dehydrated in ascending grades of ethanol, transitioned with propylene oxide and embedded in resin mixture of Embed 812 kit, cured in a 60 °C oven, and sectioned on a Leica Ultracut UC6 ultramicrotome. 1 um thick sections were collected and stained with Toluidine Blue O. 70 nm sections were collected on 200 mesh Cu grids; thin sections were stained with uranyl acetate and Reynolds lead citrate.

### RNA Extraction and qRT-PCR

RNA extraction was performed using mirVana microRNA Isolation Kit (Life Technologies) as described in manufacture’s manual. cDNAs were synthesized from DNAse-treated RNA samples and qRT-PCR was performed as described before^11^. Several internal controls were used, including *Gapdh*, *Hprt1*, *Actb*, and Sno-202 for microRNAs. List of Taqman Gene Assays used: Egr2 (Mm00456650_m1), Mpz (Mm01290519_m1), Ccnd1 (Mm00432359_m1), Hprt (Mm00446968_m1), Actb (Mm00607939_s1).

### Real-time PCR microRNA Arrays

The assays were performed on TaqMan Rodent MicroRNA A + B Cards Set v3.0 (Life Technologies) according to manufacturer’s instructions.

### Immunofluorescence

Immunohistochemistry was performed as described before^11^. Click-iT EdU Alexa Flour Imaging Kit (Molecular Probes) was used for labeling EdU.

### Xgal Staining

Samples were washed with rinse buffer (100 mM sodium phosphate pH 7.4, 2 mM MgCl2, 0.1% sodium deoxycholate, 0.2% Nonidet-P40) and then stained in rinse buffer containing 0.5 mM potassium ferricyanide, 0.5 mM potassium ferrocyanide and 1 mg/ml 5-bromo,4-chloro-3-indolyl-β-D-galactopyranoside (XGal) at 37 °C overnight, protected from light. After staining, the samples were rinsed in PBS, post-fixed them in 4% paraformaldehyde and photographed.

### Quantification and Statistics

For quantification, counts were obtained manually on three representative fields (immunofluorescence 20×, semithin 100×, electron micrographs 440×) of at least three control and three mutant animals. G-ratios were quantified using G Ratio Calculator ImageJ plugin developed by the Cellular Imaging Facility of the University of Lausanne, Lausanne, Switzerland. Two tailed, Student’s t-Test was used for calculating p-values. *Egr2* microRNA arrays were analyzed using Real Time ExpressionSuite software v1.1. (Thermo Fisher Scientific).

### Data Availability

The datasets generated during and/or analyzed during the current study are available from the corresponding author on reasonable request.
